# Holistic processing of gaze cues during interocular suppression

**DOI:** 10.1038/s41598-022-11927-w

**Published:** 2022-05-11

**Authors:** Cooper D. Jackson, Kiley K. Seymour

**Affiliations:** 1 School of Psychology, Western Sydney University, Penrith, NSW Australia; 2 The MARCS Institute, Western Sydney University, Westmead, NSW Australia; 3 Max Planck Institute for Biological Cybernetics, Tübingen, Germany

**Keywords:** Psychology, Perception, Consciousness

## Abstract

Direct eye contact is preferentially processed over averted gaze and has been shown to gain privileged access to conscious awareness during interocular suppression. This advantage might be driven by local features associated with direct gaze, such as the amount of visible sclera. Alternatively, a holistic representation of gaze direction, which depends on the integration of head and eye information, might drive the effects. Resolving this question is interesting because it speaks to whether the processing of higher-level social information in the visual system, such as facial characteristics that rely on holistic processing, is dependent on conscious awareness. The *Wollaston Illusion* is a visual illusion that allows researchers to manipulate perceived gaze direction while keeping local eye features constant. Here we used this illusion to elucidate the driving factor facilitating the direct gaze advantage during interocular suppression. Using *continuous flash suppression*, we rendered Wollaston faces with direct and averted gaze (initially) invisible. These faces conveyed different gaze directions but contained *identical eye regions.* Our results showed clear evidence for a direct gaze advantage with Wollaston faces, indicating that holistic representations of gaze direction may drive the direct gaze advantage during interocular suppression.

Eye gaze is a crucial sensory cue essential for fluid social interactions^1^, facilitating our capacity to understand the intentions and focus of others^2–6^. The ability to detect another’s gaze direction is a critical factor in the development of theory of mind^2,7^ and is reported to be impaired in neuropsychiatric conditions such as autism spectrum disorder^8^ and schizophrenia^9,10^. Research has begun to uncover the perceptual and neural mechanisms that extract information about gaze direction from face stimuli, localised in part to higher-level visual pathways in the temporal cortex^11–13^.

A reported bias to detect faces with direct gaze is well-established^14–19^. This bias is thought to reflect a biological advantage for identifying predators in the environment^1,20,21^ but given that human eyes have unique morphological characteristics (e.g., an enlarged sclera to iris ratio^22^) it may have also evolved to facilitate communication with conspecifics.

Research suggests that the direct gaze bias is evident at preconscious stages of visual processing^19,23–27^. This research commonly employs *continuous flash suppression* (CFS^28^) in which a dynamic masking pattern presented to one eye temporarily suppresses awareness of a target stimulus presented to the other eye. Using this technique, researchers have shown that faces with direct gaze gain access to conscious awareness faster than faces with averted gaze, indicating preferential preconscious processing of direct gaze by the visual system. This finding is striking because it suggests that socially relevant facial cues can be processed in the human visual system in the absence of conscious awareness.

It is important to note that our perception of gaze direction does not rest solely on information gleaned from the eyes. Rather, the percept of gaze direction is an emergent, holistic property determined by integrating multiple physical attributes^29–32^. These attributes include cues from the eye region, such as the luminance distribution produced by the position of the darker pupil and iris compared to the lighter sclera^1,33^, as well as attributes of the head, such as the orientation of the nose and overall facial symmetry^29,34^. Also, the head and eye regions interact, such that changes in the surrounding head influence the perceived gaze direction conveyed by the eyes^30^. For example, when our friend’s pupil is in an averted position this can be a strong cue that they are *not* looking at us if their face is turned directly towards us. However, if our friend’s face is turned to the side, the *same* averted pupil position can indicate that they are looking *towards* us.

The visual integration of head and eye cues is best exemplified by the Wollaston Illusion^35^ (Fig. 1). Here, identical sets of eyes are superimposed onto heads facing different directions. Despite the faces containing *identical eye regions*, the surrounding context of the head drastically alters the perceived gaze direction. Wollaston stimuli are highly useful as they allow researchers to independently manipulate the perceived direction of gaze, while keeping the key low-level stimulus characteristics constant^36–38^. Using such stimuli, there is now evidence to show that the direct gaze advantage observed in visual search and attentional cueing tasks relies on the emergent property of *perceived* gaze direction, rather than the physical properties of the eye region per se^36,38–41^*.* Similarly, sensory adaptation to Wollaston faces produces perceptual aftereffects consistent with adaptation of higher-level representations of gaze direction that depend on the integration of head and eye features, rather than adaptation to local eye features alone^37^.

**Figure 1:**
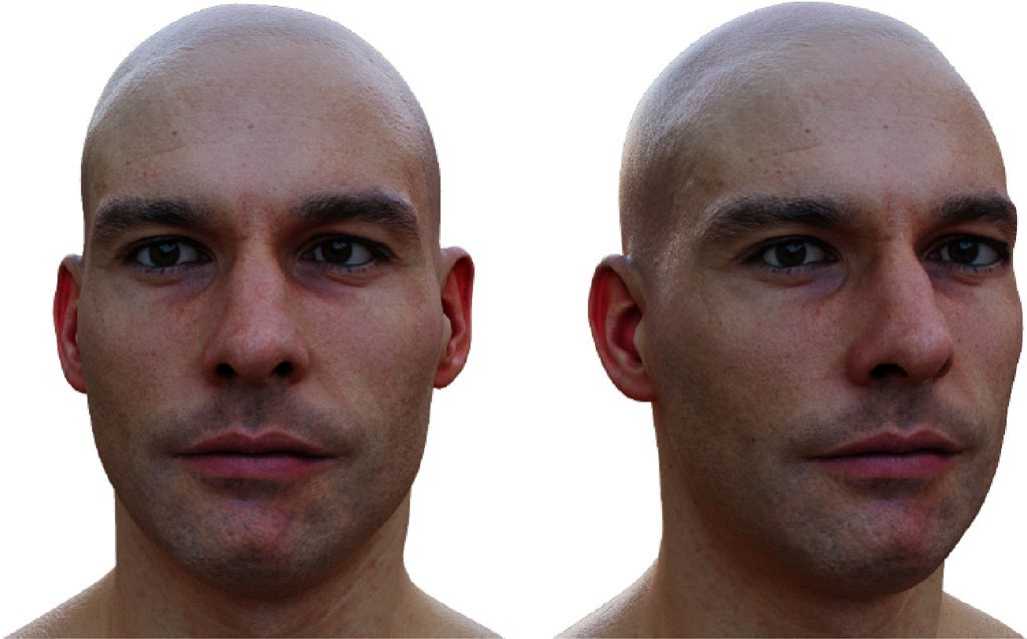
The Wollaston illusion, wherein *identical* sets of eyes (e.g., reflected in the iris position and amount of sclera visible) result in a different percept of gaze direction dependent upon the surrounding head orientation. The face images were generated with FaceGen Modeller 3.5., (https://facegen.com/).

While evidence suggests that the integration of head and eye cues occurs rapidly, it is unclear whether this form of visual processing requires conscious awareness. Similarly, we do not know whether the faster preconscious processing of faces with direct gaze observed under CFS is driven by the *physical* local properties of the eyes (e.g., differences in iris-sclera positioning) or the higher-level holistic representation of gaze direction that depends on the integration of head and eye information. In the current study, we investigated these questions by measuring the suppression of faces that elicit the Wollaston illusion using CFS. Specifically, we used Wollaston face stimuli that were *matched across conditions* in the features of their eye regions, but were perceived as looking in different directions (i.e., direct or averted gaze) due to the *conjunction* of head and eye features*.* In our design, we also controlled for differences in head features across conditions, described further in the “Methods”. We hypothesized that if integration of head and eye information occurs at a preconscious level of processing, then Wollaston faces perceived as looking directly at the observer will reach awareness significantly faster than Wollaston faces eliciting the percept of averted gaze, despite containing identical eye information. In contrast, if holistic processing of gaze direction requires conscious awareness, then no direct gaze advantage would be observed for our Wollaston stimuli given that they contain identical eye regions.

## Methods

### Ethical statement

The study was approved by the Western Sydney University Human Research Ethics Committee (H12571). All research was performed in accordance with relevant guidelines and regulations. Participants received course credit for their time and provided written and informed consent before participating.

### Participants

Forty-eight participants (10 males, 38 females) between the ages of 18 to 64 (*M* 21.65, *SD* 7.40) were recruited through Western Sydney University’s research participation system. Four participants were not run on the main experiment after providing unreliable data from the Wollaston stimulus calibration (see below). From the remaining forty-four participants, seven participants were also removed as their data suggested a non-serious attempt or a failure to understand the task (i.e., > 90% missed trials, fast responses, incorrect localisations). Data from the remaining 37 participants (6 males, 31 females, age range 18–33; *M* 20.54, *SD* 3.48) were analysed. This sample size is similar to previous CFS research^24,27,42^, and is double that required to detect a main effect of gaze direction based on prior literature^25^ (e.g., *d* = 2.04). Participants had normal to corrected vision (as assessed by the Freiburg visual acuity test^43^) and were naïve to the purpose of the study.

### Design

The current study utilised a within-subjects experimental design. Two within subject factors were manipulated: stimulus type (Standard/Wollaston) and gaze direction (Direct/Averted). The key dependent variable was stimulus suppression time (ms).

### Apparatus and stimuli

A Dell Precision 3620 tower computer running MATLAB™ (MathWorks Ltd) was used to control the experimental tasks and to record participants responses. Stimuli were presented on a 24-inch VPixx monitor (1920 × 1080-pixel resolution, 120 Hz refresh rate) using Psychtoolbox^44^. Participants viewed stimuli from a distance of 57 cm with their head stabilised using a chinrest.

**Figure 2:**
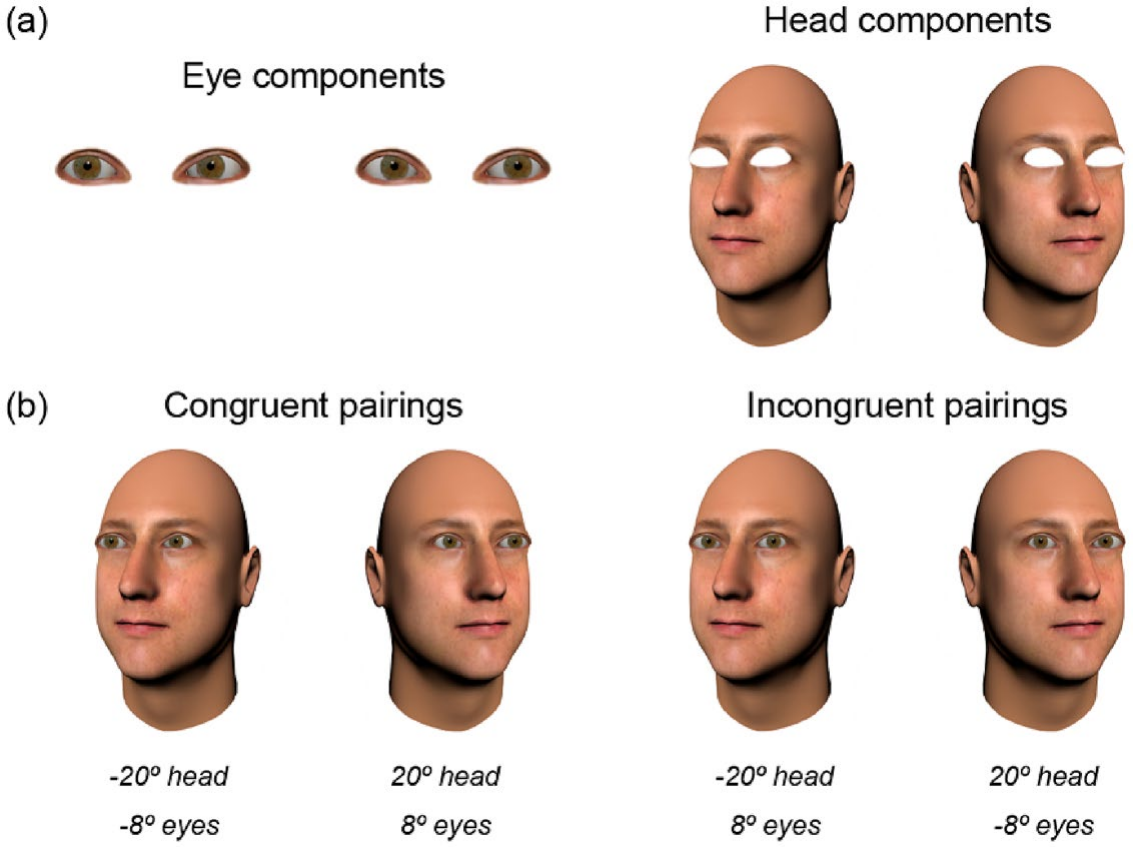
Wollaston stimuli. (**a**) Eyes isolated from a frontal head were paired with heads angled either left or right. (**b**) Congruent pairings (e.g., leftwards-angled eyes in leftwards-angled head) tended to produce a sense of averted gaze. Incongruent pairings (e.g., leftwards-angled eyes in a rightwards-angled head) tended to produce a sense of gaze directed towards the viewer. When comparing between the congruent and incongruent pairings, the stimuli are comprised of identical base components, but presented in different combinations. Hence, features like iris position, visible sclera and head rotation cues are controlled across this comparison, despite the differences in perceived direction of gaze. The face images were generated with FaceGen Modeller 3.5., (https://facegen.com/).

Stimuli were computer-generated faces presented on screen at 3.3° × 4.6°. 3D models of faces were created using FaceGen 3.5., then manipulated in the scene-based rendering program Blender 2.70. In Blender, we modelled the eyes as separate objects from the rest of the face, allowing head orientation and gaze direction to be precisely controlled. Eye deviations reported here are in viewer-centred coordinates. For example, when eyes are directed 0, they are looking directly at the viewer (i.e., the camera the image is rendered from). The experiment included two types of stimuli: Wollaston stimuli and Standard stimuli. Each stimulus type was presented with both direct and averted gaze, for both rightward and leftward oriented heads. Wollaston stimuli comprising the same base eye-region and head-region components (Fig. 2) were constructed following the method outlined by Palmer and Clifford^37^. This involved using Adobe Photoshop to merge eye regions and head regions from different images. Direct and averted gaze conditions were created by pairing either congruent or incongruent conjunctions of these base components. Congruent eye and head pairings (e.g. rightward eyes in a rightward head) resulted in face stimuli perceived as displaying averted gaze. Incongruent pairings of the same head and eye components (e.g. rightward eyes in a leftward head) resulted in face stimuli that were perceived as displaying direct gaze. The use of such stimuli allowed us to examine the processing of different gaze directions while keeping physical low-level properties of the stimuli matched overall across conditions (i.e., direct and averted conditions were each made up of the identical eyes directed left and right and the identical heads rotated left and right, but presented in different combinations).

**Figure 3:**
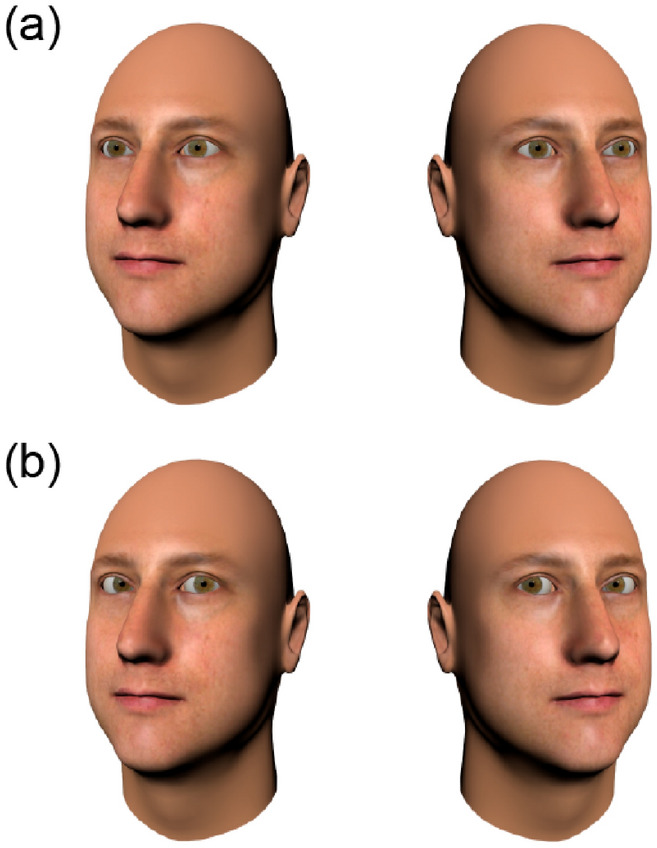
Example of the Standard stimuli. These stimuli have gaze directed away from the viewer (**a**) or towards the viewer (**b**). The eyes in these faces differ in low-level *physical* cues to gaze direction (e.g., amount and position of visible sclera). The heads and eyes on the top row (**a**) are deviated at an angle of 20° relative to the viewer, whereas the bottom row contains stimuli with heads rotated at 20° and eyes deviated at an angle of 0° relative to the viewer. The face images were generated with FaceGen Modeller 3.5., (https://facegen.com/).

Standard stimuli were created with the same face identities and head rotations used to create the Wollaston stimuli (Fig. 3). However, the eye regions were not identical across averted and direct conditions. Instead, analogous to previous research^23,27,45^, we allowed for low-level differences in visible-sclera to remain in the stimuli. Specifically, we used the same eye deviations employed in previous studies; faces with direct gaze had an eye deviation of 0° and averted gaze had an eye deviation of 20° or − 20°. The inclusion of these stimuli in our experiment allowed us to verify the presence of a direct gaze advantage with computerized faces.

Six face identities were used for both the Wollaston and Standard stimuli. All stimuli were comprised of heads rotated laterally 20° leftwards or rightwards. Unique eye deviations were determined for each participant prior to running the CFS task (see Pre-test eye deviation calibration task below) to ensure the appropriate eye deviation was incorporated into the Wollaston stimuli to elicit the strongest illusion (i.e., where an incongruent head-eye pairing was subjectively perceived as direct and a congruent pairing was perceived as averted).

### Procedure

#### Pre-test eye deviation calibration

Prior to the CFS experiment, each participant completed a calibration task to determine the optimal magnitude of eye deviation required to elicit the Wollaston illusion. Previous research has shown that there are considerable individual differences in gaze perception^18,46–48^. This step ensured that, for each participant, the stimuli used were subjectively perceived as averted gaze for congruent pairings of head and eye direction and perceived as direct gaze for incongruent pairings of head and eye direction. Following a similar approach to Palmer and Clifford^37^, participants viewed Wollaston stimuli and judged the perceived gaze direction from faces with eye deviations of 4°, 6°, 8°, 10° or 12°. Participants saw one stimulus at a time and were required to report whether they perceived the gaze direction as left, direct, or right. Stimuli were presented for 1 s in each trial, and participants completed 24 trials of each head/eye combination. The viewing distance, size and placement (2.2° to the left or right of fixation, presented randomly across trials but balanced across eye deviations) of stimuli were the same as that used in the subsequent CFS experiment^34^. On completion, data for each participant was visually inspected to find the angle of eye deviation associated with the cleanest pattern of direct percepts for incongruent head/eye combinations and averted percepts for congruent head/eye combinations. This eye deviation was then selected for that subject as the optimal gaze deviation and used to create their Wollaston stimuli for the CFS task. For example, if a participant’s optimal gaze direction was 8°, the deviations for the direct and averted Wollaston Stimuli was 8° or − 8°. Participants were precluded from performing the CFS task, if none of the tested eye deviations produced a consistent perceptual distinction between the congruent and incongruent head/eye combinations.

#### Continuous flash suppression (CFS)

We closely followed the CFS method used by Stein et al.^27^. Participants viewed a dichoptic display through a mirror stereoscope. Two red squares (11° × 11°) were presented on a uniform grey background. When viewed through the mirror stereoscope, the left eye saw only the left square and the right eye saw only the right square (confirmed prior to testing by asking participants to report their percept when viewing the stimulus monocularly). When binocularly fused, only one square was apparent to the participant. Fusion contours (width 0.8°) comprising black and white pixels helped to maintain binocular fusion throughout the task.

**Figure 4:**
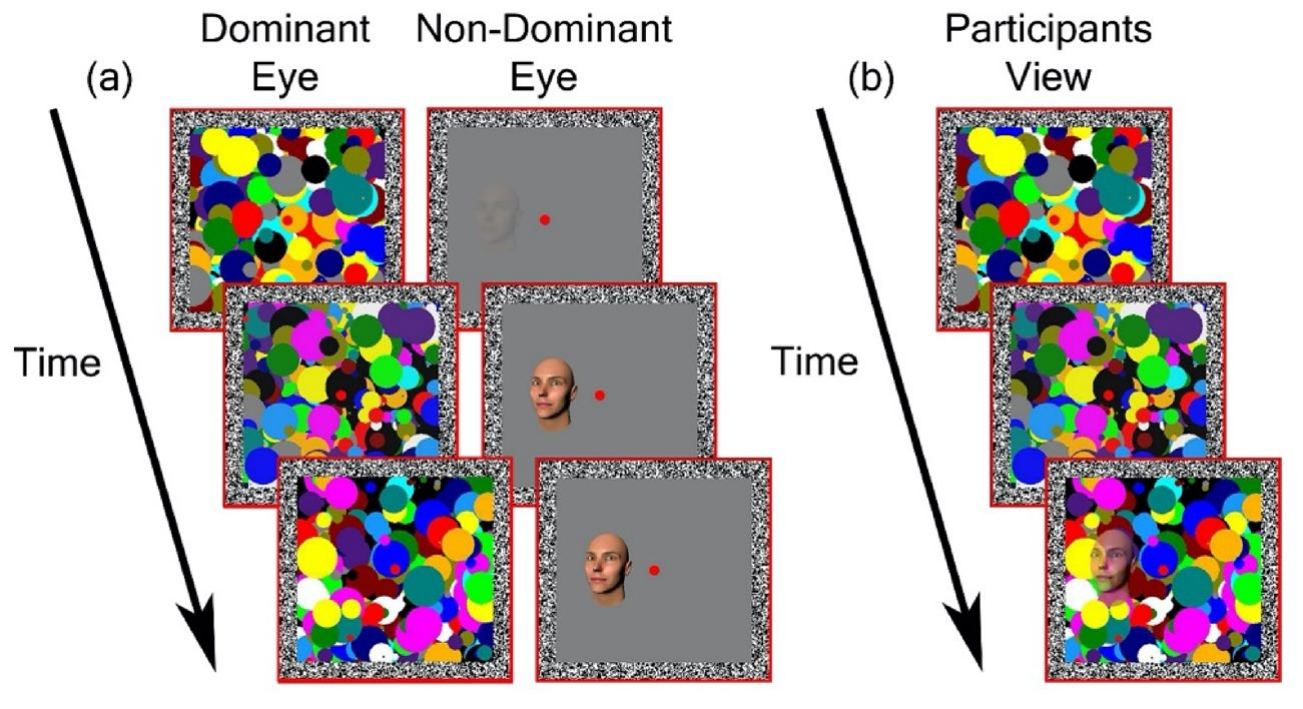
(**a**) A schematic of an example CFS trial. A high contrast flashing mask was presented to the participant’s dominant eye, while a face stimulus (here Standard Direct) was presented to the non-dominant eye. (**b**) A schematic example of a participant’s view during the task. The binocular presentation results in the temporary suppression of the face stimulus from conscious awareness. Participants are instructed to respond as quickly and as accurately as possible when any aspect of the face becomes visible.

Figure 4 illustrates a single trial of CFS task. Each trial began with a 1 s presentation of the red squares, fusion contours, and a central fixation dot (0.5°). Next, a face was presented to the non-dominant eye (confirmed using the near convergence test^49^), initially presented at a contrast of 0% which increased linearly to 100% within a period of one second window. At the same time, a high contrast coloured mask flashing at 10 Hz was presented at full contrast to the participant’s dominant eye. This resulted in a temporary suppression of the face stimulus, rendering it invisible to the participant. Face stimuli were presented 2.2° to the left or right of the fixation with their centre being aligned vertically with the central dot. This location was randomly selected on any given trial but was balanced across conditions.

Participants were required to maintain fixation throughout the experiment. They were instructed to indicate with the left or right arrow key whether the face appeared to the left or right of the fixation dot. They were instructed to respond as quickly as possible when any aspect of the face became visible and were not required to make judgements about the stimulus. Once a response was made, this initiated the next trial. A maximum trial length was 10 s. Response latencies greater than 8 s were excluded from the analysis, as at this stage of the trial the contrast of the flashing mask decreased linearly to 0% within a period of one second window. Trials with response latencies quicker than 300 ms were also removed, as these were likely to reflect a false response^50,51^. Participants completed a total of 576 trials wherein each of the 4 conditions (i.e., Wollaston Direct, Wollaston Averted, Standard Direct, Standard Averted) were presented 144 times in a completely randomised manner. A break was provided halfway through the task. Prior to testing, participants completed a number of practice trials to confirm visual fusion was achieved and that the task instructions were understood. Practice trials were not analysed. Only trials with correct responses were submitted to statistical analysis.

### Statistics

**Figure 5:**
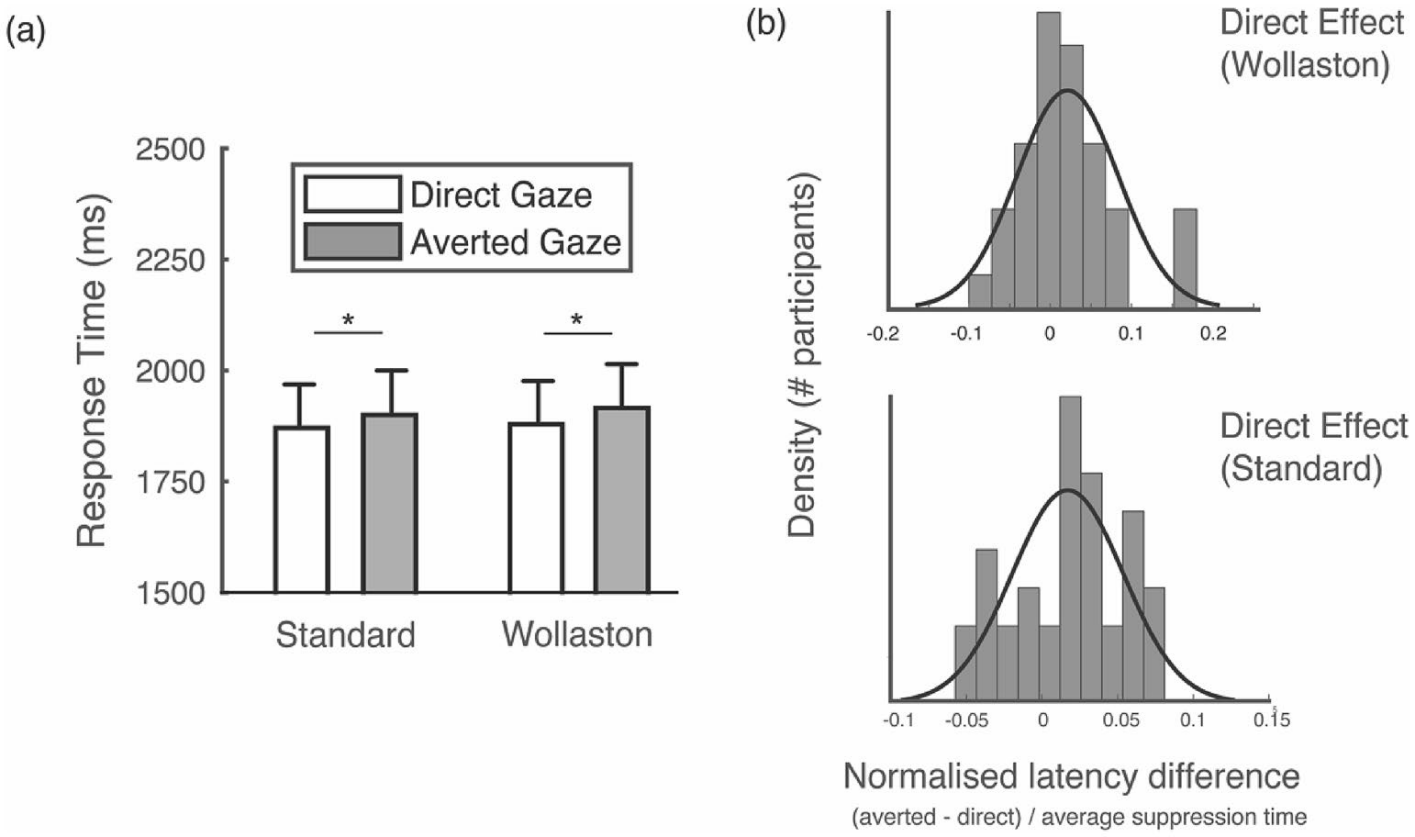
Mean suppression times for each condition (**a**). Error bars denote ± standard error. *(p < 0.05). (**b**) Sample distributions of the direct effect (i.e., the extent to which faces with direct gaze are detected faster than faces with averted gaze) are reported as latency normalised mean differences in suppression times between averted and direct conditions for Wollaston stimuli and Standard stimuli. Values above zero indicate a direct gaze effect. Note, not all participants show the direct effect with Wollaston or Standard stimuli.

Suppression times were calculated as the time taken to localise the face after it appeared at full contrast (i.e., a second after the start of the trial) and used as an index of stimulus potency in reaching conscious awareness. Each individual’s mean suppression times were normalised to their mean to account for individual differences and positive skew^19,52^ (i.e., mean suppression times for each condition were divided by the participant’s mean across all conditions). All statistical analyses were conducted on the normalised data, but Fig. 5a shows raw data in milliseconds to facilitate an intuitive interpretation of the results (also see Supplementary Information for analysis on raw suppression times).

In addition to classical statistics, we report the results of a Bayesian repeated-measures ANOVA computed with default priors. We report Bayes factors for the inclusion of each effect in the ANOVA model (BF_incl_), which represent the likelihood of the data given the model(s) that include the effect relative to matched models that lack the effect. We also report Bayesian paired samples *t*-tests, computed with a default Cauchy prior scale of 0.707. We report one-tailed BF_10_ values for *t*-tests to quantify evidence for the alternative hypothesis over the null hypothesis. These analyses were conducted using JASP version 0.12.2 (JASP Team, 2020). The method for these analyses followed recommendations in Keysers et al.^53^.

## Results

### Perception of Wollaston images

To control for individual differences in direct gaze perception, the Wollaston stimuli shown during the CFS task were tailored to individual participants. For the majority of the 48 participants tested, the optimal magnitude of eye deviation to elicit the Wollaston illusion was either 6° or 8°. With these deviations, participants consistently judged the faces as exhibiting direct gaze for incongruent head/eye combinations (M 80% SD 15%) and averted gaze for congruent head/eye combinations (M 99% SD 14%). Four participants were precluded from the CFS experiment based on results from the pre-test eye deviation calibration task, as none of the tested eye deviations produced a consistent perceptual distinction between the congruent and incongruent head/eye combinations.

### Continuous flash suppression

Data from seven participants were removed from the analysis due to a high proportion (i.e., > 90%) of invalid trials. For the remaining 37 participants, accuracy data is as follows: Wollaston direct (M 96.82%, SD 4.57%), Wollaston averted (M 97.35%, SD 4.40%), Standard direct (M 97.33%, SD 4.46%), Standard averted (M 97.76%, SD 4.40%). A 2 × 2 repeated measures ANOVA conducted on these data revealed a significant difference in accuracy between Wollaston and Standard stimuli (F(1, 36) = 4.776, p = 0.035, η^2^ = 0.038) with slightly more correct trials being observed with Standard stimuli. Accuracy also differed between averted and direct trials (F(1, 36) = 4.479, p = 0.041, η^2^ = 0.043), with slightly more averted trials being observed. Importantly, however, there was no interaction between gaze direction and stimulus type (F(1, 36) = 0.056, p = 0.815) that could pose a potential confound with our experimental design and interpretation of the suppression time results.

Normalised mean suppression times were calculated for Wollaston and Standard conditions for both direct and averted gaze stimuli for each participant. In testing whether the integration of head and eye information occurs at a preconscious level of processing, we sought evidence for a direct gaze advantage for both Wollaston and Standard face stimuli. We conducted a 2 × 2 repeated measures ANOVA with gaze direction and stimulus type as factors. We found no main effect for stimulus type, F(1, 36) = 2.170, p = 0.15, η_p_^2^ = 0.057, BFincl = 0.42, indicating a good match in low-level properties between our Wollaston and Standard stimuli. Consistent with our hypothesis, our results revealed strong evidence for a main effect of gaze direction, *F*(1, 36) = 7.91, *p* = 0.008, η_p_^2^ = 0.18, BF_incl_ = 82.01. We found no evidence for a significant interaction, *F*(1, 36) = 0.308, *p* = 0.582**,** η_p_^2^ = 0.002, BF_incl_ = 0.27 (Fig. 5a), indicating that the direct gaze advantage is not significantly influenced by stimulus type. Post-hoc paired *t*-tests (one-sided) verified a difference in suppression times between direct and averted gaze with Standard stimuli, *t*(36) = 2.50, *p* = 0.009, *d* = 0.410, BF_10_ = 5.17. Consistent with prior research using real-face stimuli^23,25,27^, faces with direct gaze became visible significantly faster (*M* 1878 ms, *SD* 544) than averted gaze faces (*M* 1907 ms, *SD* 549). Similarly, we found a significant difference between direct (*M* 1886 ms, *SD* 536) and averted gaze conditions (*M* 1922 ms, *SD* 548) with Wollaston stimuli, *t*(36) = 2.07, *p* = 0.023, *d* = 0.340, BF_10_ = 2.31.

Because normality assumptions were violated in our data we also ran statistical analyses on latency normalised difference in suppression times between averted and direct for Wollaston stimuli and Standard stimuli^54^. For each participant and each stimulus type (Wollaston or Standard), we calculated the difference in mean suppression time between averted and direct gaze divided by the participants’ overall mean suppression time. One sample t-tests (one-sided) of these data showed a significant direct effect for both Wollaston (t(36) = 2.070, p = 0.023, d = 0.340, BF10 = 2.311, normalised mean suppression time difference = 0.021, 95% CI [0.06, ∞]) and Standard stimuli (t(36) = 2.491, p = 0.009, d = 0.410, BF10 = 5.170, normalised mean suppression time difference = 0.015, 95%, CI [0.125, ∞]. We plot sample cumulative distributions of the direct effect for Wollaston and Standard face stimuli (Fig. 5B).

In the current study, the difference in detection times between direct and averted gaze were around 29–36 ms on average. This is similar to at least one previous study that used CFS to investigate the direct gaze effect^24^, though some past studies have tended to find effects closer to ~ 0.5–1 s^25,27^. This may be related to suppression times being generally shorter across conditions in the current study compared to these previous studies. Shorter suppression times may have occurred in the current study due to the use of coloured rather than greyscale face stimuli. Moreover, it has been shown that similarity in visual properties between mask and target stimuli can influence suppression times^55^. The majority of previous studies reporting a direct gaze advantage with larger effect sizes use greyscale faces and colour masks. Our experiment deviated from this also, which could have led to the shorter suppression times observed in our study.

## Discussion

The aim of the current study was to elucidate whether the integration of head and eye information occurs outside of conscious awareness. Using CFS, we tested whether the direct gaze advantage during interocular suppression^25–27^ is driven by the holistic representation of gaze direction that requires the integration of head and eye cues or whether it depends on local differences in the physical properties of the eyes. In particular, we used the Wollaston illusion, in which identical eye regions are perceived as looking in different directions depending on the orientation of the head (Fig. 1). We created stimuli with different conjunctions of head and eye features that result in different perceived directions of gaze, whilst maintaining *identical* physical eye region characteristics across direct and averted gaze conditions. We hypothesized that if integration of head and eye information occurs at a preconscious level, then the Wollaston conjunctions perceived as looking direct (i.e., incongruent head and eye deviations) would gain access to awareness over Wollaston faces perceived as looking away (i.e., congruent head and eye deviations), despite the identical eye features that these stimuli contained. We found clear evidence for a direct gaze advantage with Wollaston faces during CFS, suggesting the integration of head and eye information does not require conscious awareness. Moreover, our study suggests that the prioritised unconscious processing of direct gaze observed in previous studies might be driven more by a holistic representation of gaze direction rather than low-level morphological differences in eye regions.

It has previously been shown that humans are hyper-sensitive to low-level sensory signals that convey the attentional focus of our conspecifics^16,45,56^. For instance, it has been reported that young infants can differentiate direct and averted gaze by relying on physical information such as motion of the pupil or the contrast distribution within the eye socket^57,58^. Also, adult humans are sensitive to slight changes in the size, luminance or contrast of the sclera or the iris, which can significantly influence perceived gaze direction^33,59–61^. The results of our study, which removed these low-level differences between direct and averted gaze stimuli, suggest that such information is not necessary for driving the direct gaze advantage during interocular suppression. Instead, our data indicate that at unconscious stages of visual processing, the visual system may be sensitive to direct gaze as an integrated perceptual property, independent of the specific low-level features that signal it.

In the original study by Stein et al.^27^, low-level differences in eye regions were manipulated by inverting the contrast of the eyes (i.e., making the sclera dark and iris light). This manipulation retained local contrast differences but abolished the direct gaze advantage during interocular suppression^27^. This result could be taken as evidence for a reliance on low-level eye region characteristics being critical for the early unconscious processing of gaze direction. To the best of our knowledge, the current study is the first to assess the direct gaze advantage using Wollaston face stimuli. Our approach allowed the perceived direction of gaze to be disassociated from low-level stimulus characteristics. In particular, the Wollaston faces that we compared across were composed of identical eye regions and identical head regions, but presented in different combinations that resulted in different perceived directions of gaze. Thus, no differences in sclera shape and position were apparent in our stimuli. By using Wollaston stimuli we were able to test for a direct gaze advantage across stimuli that differed in the perceived direction of gaze (i.e., relying on holistic processing of the face), while matching all low-level morphological features in the eye region. Hence, our finding of a direct gaze advantage during CFS is consistent with studies showing that early fundamental mechanisms involved in the perception and automatic orienting to another’s gaze direction may rely on the holistic representation of gaze direction, rather than the local eye region per se^30,36–41,62,63^*.*

The detection of eye contact has been proposed to initially recruit a fast-subcortical pathway involving the superior colliculus, amygdala, and pulvinar^27,64^. This pathway is believed to be hardwired and to operate outside of conscious awareness^65–67^ and hence might underlie the processing of gaze direction during CFS^28^. While there is evidence that higher cortical areas such as the superior temporal sulcus (STS) carry information about gaze direction that is invariant to head and eye deviations, the contribution of subcortical areas to head and eye integration has been elusive. In the current study, we show evidence suggesting the integration of head and eye information occurs at an unconscious level of processing. Because CFS is thought to disrupt processing in cortex^67,68^ we therefore speculate that a subcortical locus may carry the integrated signal of head and eye information that leads to the prioritized and rapid detection of direct gaze. However, it should be noted that although our study assumes differences in suppression times to reflect differences in the unconscious processing that our stimuli receive while being suppressed, debates highlight the possibility that such differences may reflect disparities at very early conscious stages of processing^41,66^. Some b-CFS studies base their conclusions about unconscious processing on a comparison with a non-CFS control condition. In designing our experiment, we did not include such a control as we believe it lacks validity. For instance, it has been shown that such control conditions have rather poor sensitivity for measuring differences between conditions and fail to mimic the rather unique perceptual experience under CFS (see: Stein and Peelen, 2021)^69^. Namely, these control conditions differ from b-CFS conditions in subjective perceptual experience, target appearance, perceptual uncertainty, and target predictability. In a recent CFS study, Stein and Peelen^69^ advocate for use of an additional discrimination task to rule out conscious processes as a cause of suppression time differences. Their data showed that despite participants being unable to report the orientation of a detected face, there were differences between upright and inverted faces reflected in suppression times. We did not employ this type of control. However, future extensions of our work with this new detection-discrimination approach to CFS may provide additional support for the integration of head and eye information occurring outside of awareness. Importantly, though, the current results as they stand provide a clear indication that the integration of eyes and head information occurs rapidly and that detection of direct eye gaze (based on the integration of head and eyes) is prioritised by the visual system. This supports other research using the Wollaston illusion that shows a reflexive orienting response to Wollaston faces with direct gaze in infants, suggesting that the mechanism for integrating head and eye information is hardwired and automatic^62^.

In our study, the use of a calibration task verified that the Wollaston stimuli used in the CFS experiment were consistently perceived as looking in different directions (categorised as either direct or averted) when presented in the absence of a CFS mask. This is an important aspect of our design, as large individual differences in gaze perception have been reported in the literature^46^. In particular, gaze perception and the automatic orienting to direct gaze has been shown to be altered in conditions such as schizophrenia and autism spectrum disorder^10,70–73^. Future research employing our approach may help to elucidate whether these anomalies result from perturbations in the early integration of head and eye information. In addition, our data reveal that gaze direction may be represented holistically at an early preconscious stage of processing. Holistic processing is a distinct feature of facial processing^74,75^. However, research suggests that the integration of some individual features into a interdependent representation may involve a two-stage process requiring conscious awareness (e.g., internal and external face information^76^, emotional facial expression and gaze direction^77^). Thus, future research to determine the type of social information that is integrated rapidly at the initial preconscious stages of visual processing will provide important insight into the mechanisms of holistic processing and social perception.

## Supplementary Information

Supplementary Information.
