# Supplementary material for: Holistic processing of gaze cues during interocular suppression
Source: Sci Rep. 2022 May 11;12:7717. doi: 10.1038/s41598-022-11927-w (PMC9095640; doi:10.1038/s41598-022-11927-w)
Supplement: Supplementary file 1 — Supplementary Information. [file 41598_2022_11927_MOESM1_ESM.docx]

**"Holistic processing of gaze cues during interocular suppression"**

**Supplementary Material.**

**Analysis of RAW suppression time data.**

We conducted a 2 x 2 repeated measures ANOVA with gaze direction and stimulus type as factors.

| **Within Subjects Effects** | | | | | | | | | | | | | |
| --- | --- | --- | --- | --- | --- | --- | --- | --- | --- | --- | --- | --- | --- |
|  | | **Sum of Squares** | | **df** | | **Mean Square** | | **F** | | **p** | | **η²_p_** | |
| Stimulus Type |  | 4872.859 |  | 1 |  | 4872.859 |  | 1.073 |  | 0.307 |  | 0.029 |  |
| Residual |  | 163435.397 |  | 36 |  | 4539.872 |  |  |  |  |  |  |  |
| Gaze Direction |  | 39357.499 |  | 1 |  | 39357.499 |  | 7.975 |  | 0.008 |  | 0.181 |  |
| Residual |  | 177667.479 |  | 36 |  | 4935.208 |  |  |  |  |  |  |  |
| Stimulus Type ✻ Gaze Direction |  | 339.194 |  | 1 |  | 339.194 |  | 0.098 |  | 0.756 |  | 0.003 |  |
| Residual |  | 124995.935 |  | 36 |  | 3472.109 |  |  |  |  |  |  |  |
|  | | | | | | | | | | | | | |
| *Note.*  Type III Sum of Squares | | | | | | | | | | | | | |
| \| **Paired Samples T-Test** \| \| \| \| \| \| \| \| \| \| \| \| \| \| \| \| \| \| \| --- \| --- \| --- \| --- \| --- \| --- \| --- \| --- \| --- \| --- \| --- \| --- \| --- \| --- \| --- \| --- \| --- \| --- \| \|  \| \| \| \| \| \| \| \| \| \| \| \| \| \| **95% CI for Cohen's d** \| \| \| \| \|  \| \|  \| \|  \| \| **t** \| \| **df** \| \| **p** \| \| **Cohen's d** \| \| **Lower** \| \| **Upper** \| \| \| Wollaston Averted RT (eye and head congruent) \|  \| - \|  \| Wollaston Direct RT (eye and head incongruent) \|  \| 1.997 \|  \| 36 \|  \| 0.027 \|  \| 0.328 \|  \| 0.048 \|  \| ∞ \|  \| \|  \| \| \| \| \| \| \| \| \| \| \| \| \| \| \| \| \| \| \| Note.  Student's t-test. \| \| \| \| \| \| \| \| \| \| \| \| \| \| \| \| \| \| \| Note.  All tests, hypothesis is measurement one greater than measurement two. \| \| \| \| \| \| \| \| \| \| \| \| \| \| \| \| \| \| | | | | | | | | | | | | | |
